# T-cell-lymphoma presented as a solitary subcutaneous mass in the ventral cervical region of an adult llama- diagnostic and treatment

**DOI:** 10.1186/s12917-022-03158-y

**Published:** 2022-02-01

**Authors:** Julia Schoiswohl, Cassandra Eibl, Rhea Haralambus, Karoline Lipnik, Katrin Schieder, Sonja Franz

**Affiliations:** 1 Department for Farm Animals and Veterinary Public Health, University Clinic for Ruminants, University of Veterinary Medicine Vienna, Veterinärpl. 1, 1210 Vienna, Austria; 2 Department/Hospital for Companion Animals and Horses, University Clinic for Ruminants, University of Veterinary Medicine Vienna, Veterinärpl. 1, 1210 Wien, Austria; 3 Institute of Pathology, Department of Pathobiology, University of Veterinary Medicine Vienna, Veterinärpl. 1, 1210 Vienna, Austria; 4 Diagnostic Imaging, Department for Companion Animals and Horses, University of Veterinary Medicine Vienna, Veterinärpl. 1, 1210 Vienna, Austria

**Keywords:** Endoscopy, Fine needle aspiration, Lymphoma, Pathohistological examination, Radiographs, South American Camelids, Surgery, Ultrasound

## Abstract

**Background:**

Neoplasm in South American camelids (SAC) are commonly described. The most frequently reported type of neoplasm are lymphomas and difference in the age suffering from lymphomas of and llamas is seen.

This report describes a case of a solitary lymphoma in a 5 years and 9 month old llama mare displaying the approach of diagnostic imaging and successful surgical treatment.

**Case presentation:**

The llama was referred to the clinic for dyspnoea and inspiratory abnormal respiratory sounds. The clinical examination comprised blood cell count, ultrasonographic and radiographic examinations, endoscopy and fine needle aspiration cytology of a mass detected in the mid cervical region. The mass was surgically removed. Histopathological examination of the surgically removed mass diagnosed a malignant T-cell- lymphoma. According to the results of the clinical, ultrasonographic and radiographic examinations no tumor invasion was apparent in distant organs and the llama was discharged from the clinic seven days after surgery.

**Conclusion:**

Lymphoma has been reported to be the most common neoplasia in camelids and are more often described in young alpacas and in adult llamas. To the author´s knowledge the case presented here is the first that described a broad panel of diagnostic tools including ultrasound, radiographs, endoscopy, fine needle aspiration cytology and histopathoogical examination as well as a successful surgical treatment of a solitary lymphoma in camelids.

## Background

In South American camelids (SAC) neoplasms are commonly seen [1–6]. The most frequently reported type of neoplasias are lymphomas. In literature, a difference in the age of alpacas (2 years of age and less) and llamas (5–7 years) is described suffering from lymphomas [1, 4, 7–10]. Marchionatti et al. [11] reported a similar case of a solitary tracheal lymphoma in an adult alpaca. This alpacas was also referred for dyspnoea and inspiratory noise and a clinical examination, endoscopy, ultrasound, radiographs and computed tomography was performed. In difference to the case presented here, the owner requested euthanasia due to bad prognoses. The purpose of this case report is to show the diagnostic work up and surgery of a llama with a malignant round cell tumor (T-cell-lymphoma) and the immunohistochemical findings.

## Case presentation

### History

A 5 year and 9 month old, 170 kg weighing (body condition score (BCS) 3.5) domestic llama mare was referred to the University Clinic for Ruminants at the University of Veterinary Medicine Vienna due to dyspnoea and inspiratory noise for one week. An esophageal obstruction had been suspected by the referring veterinarian, however administration of spasmolytics and passing of a tube had not improve the symptoms.

### Clinical findings

The mare was bright, alert, in good general condition and had a BCS of 3.5 out of 5. In the middle third of the ventral cervical region, a protruding mass was present. The mass was 10 × 8x3 cm, partially firm and fluctuant on palpation, slightly painful and the palpable local temperature was mildly increased. The overlying skin appeared normal. No further masses or external lymph node enlargement was detectable. There were no signs of dysphagia. Besides these findings all other vital parameters were within normal ranges. Blood cell count showed mild leukocytosis, neutrophilia, lymphopenia and eosinopenia. Haematocrit (39.80%; norm 27.00–39.0%) and MCV (32.3 fL; norm 24.8–29.3fL) were mildly increased, MCHC (33.9 g/dL; norm 39.6–43.0 g/dL) was mildly decreased. TWBC was increased (23,690.0/µL; norm 8700.0–17,500.0/ µL). Segmented neutrophils were mildly increased (92.4%; norm 42.4–76.4; 21,889.56/ µL; norm 4600.00–11,400.00/ µL), Lymphocytes (3.0%; norm 7.0–32.5%; 710.70/ µL; norm 900.00–1100.00/ µL) and Eosinophils (0.4%; norm 5.0–27.7%; 94.76/ µL; norm 500.00–3700.00) were moderate decreased. All other values were within normal limits.

### Diagnostic imaging examinations

#### Endoscopic examination

Flexible endoscopy (endoscope with 1.5 m length and 8 mm diameter; Karl Storz Endoskop Austria GmbH) was performed in the sedated llama (xylazine (Sedaxylan; Dechra Veterinary Products; Dornbirn; AUT), butorphanol (Butomidor; Richter Pharma AG; Wels; AUT), each 0.2 mg/kg, IM) via ventral nasal meatus in order to view the esophagus. No pathological findings were detected over the entire length of the esophagus, the mucosa was whitish and smooth, peristaltic movements were seen. Endoscopic examination of the trachea and airways was inconspicuous.

#### Ultrasonography

**Fig. 1:**
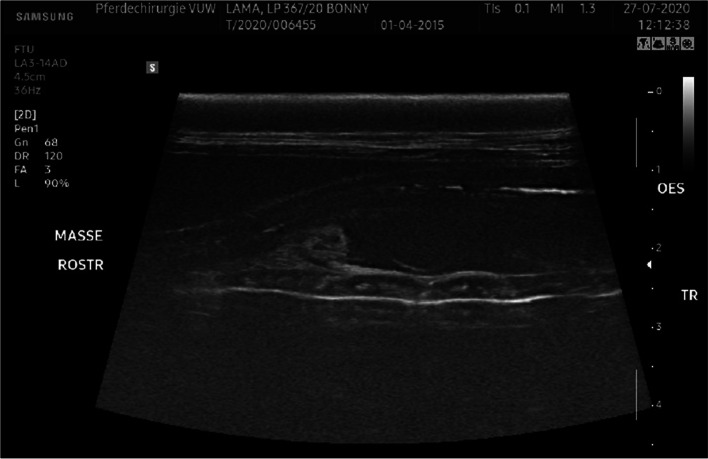
Ultrasonographic image of the mass in longitudinal view. Cranial is to the left. Upper arrow: gas-filled esophagus, star: mass, lower arrow: trachea

**Fig. 2:**
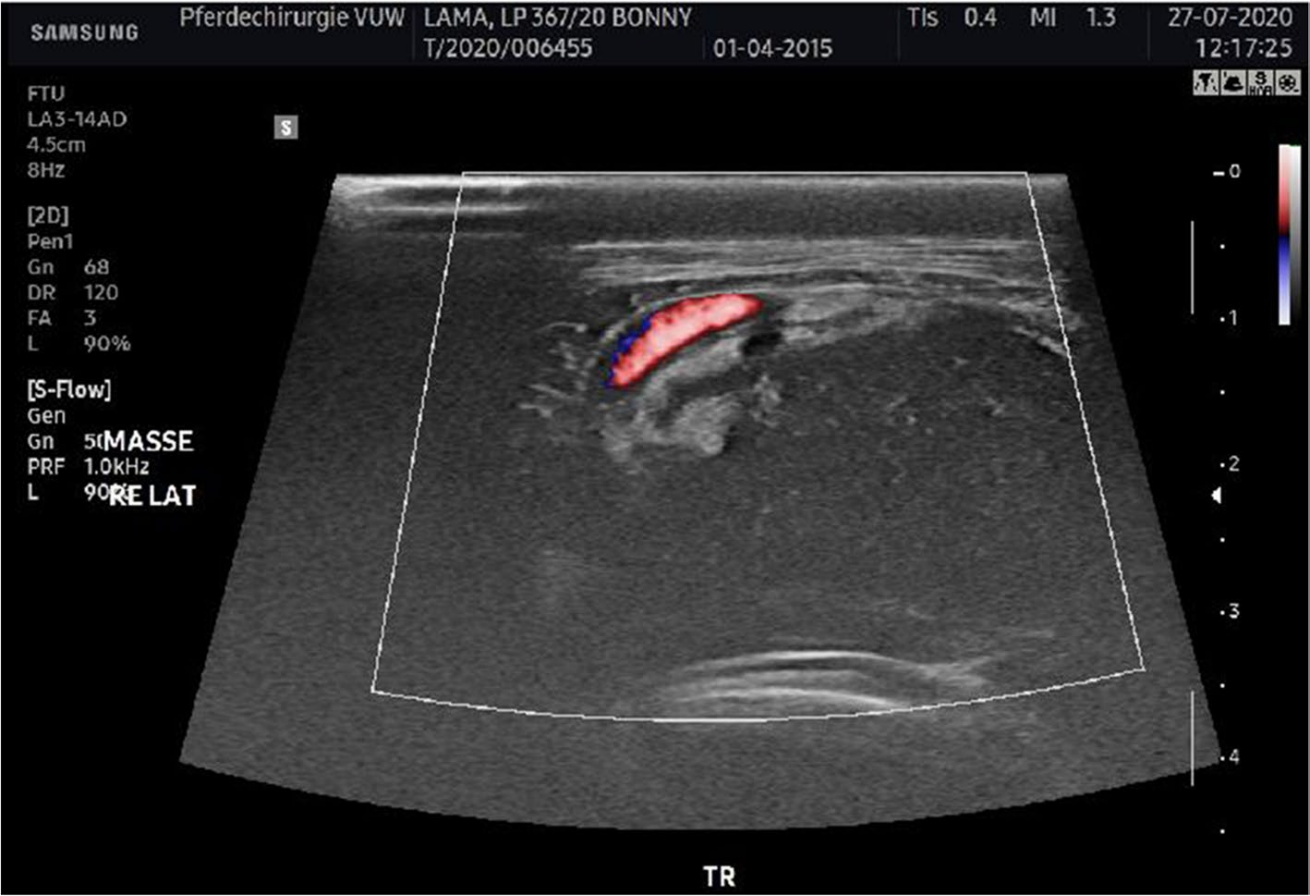
Ultrasonographic image of the mass in transverse view. Lateral right is on the left. Arrow: mass, the carotid artery is delineated in the left upper corner of the image with colour flow Doppler

Ultrasonography was performed with a 14 MHz linear probe used in trapezoid fashion (Samsung HS60). The mass presented subcutaneously and well demarcated from the surrounding tissue with heterogenic echogenicity and a firm capsule (Fig. 1). There were no signs of communication with the trachea or esophagus. The jugular vein and carotid artery were detectable but there was no evidence for vascularization of the mass itself (Fig. 2). A neoplasia or abscess formation was suspected.

#### Radiology

**Fig. 3:**
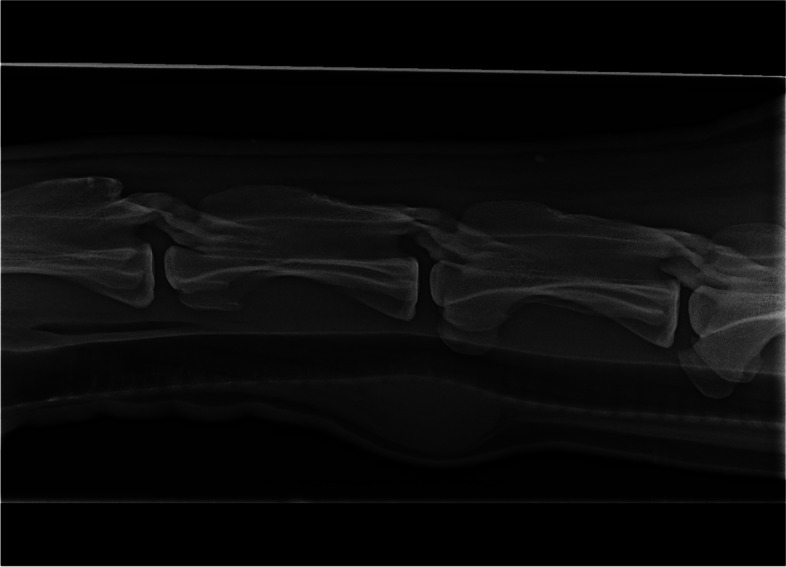
latero- lateral radiograph of the mid to caudal cervical third. Cranial is to the left. Caudal C3 to cranial C6 are depicted. There is a partially well delineated soft tissue dense mass ventral (*) to the junction of C4 to C5 with a mild mass effect to the trachea

Latero-lateral and dorso 35°left lateral- ventrolateral oblique radiographs of the mid to caudal cervical third were performed by the use of a CR System FCR IP Typ CC Pb 24 × 30 cm (Fujifilm, Tokyo, Japan) and a mobile radiographic machine (Mobilett XP, 70 kV, 6,3mAs, FFD = 1 m Siemens Healthcare, Eschborn, Germany). A ventrally well and dorsally ill defined, homogenic mass of soft tissue density could be depicted. A mild mass phenomenon to the left ventral aspect of the trachea was visible. All other structures were within normal ranges. Latero-lateral radiographs (90 kV; 6.3mAs; FFD = 1.20 m) of the thorax showed no pathological signs related to the mass (Fig. 3).

#### Surgery

The llama was put under general anesthesia in ventral recumbency with the head and neck suspended from the ceiling in a moderate extended position. After routine aseptic preparation of the surgical field, the mass was punctured under ultrasonographic control with an 18 g needle but no fluid could be aspirated.

A skin incision with approximately 12 cm in length was performed slightly right to the paramedian. With a combination of sharp and blunt preparation the mass was dissected from the surrounding tissue. The ventral part of the mass was well delineated, however some adhesions of fibrous and muscle tissue were present at its dorsal aspect. A small artery branching off the right commune carotid artery entering the mass was identified and transected after ligation. Analogous the venous blood supply was identified as a small branch of the left jugular vein which was also transected after cauterization. The mass showed a firm even surface and could be removed in toto. After cauterization of minor bleeding the incision was closed in three layers. The deep connective and subcutaneous tissue were closed in simple continuous fashion with No. 2/0 gluconate (Monosyn®), for the skin a combination of vertical U and simple interrupted sutures with polypropylene No. 0 and 1 was used.

A stent bandage was sutured over the surgical site and covered with adhesive bandage material. The incision healed uneventfully and the sutures were removed 12 days after surgery.

### Histopathological and immunohistochemical findings

**Fig. 4:**
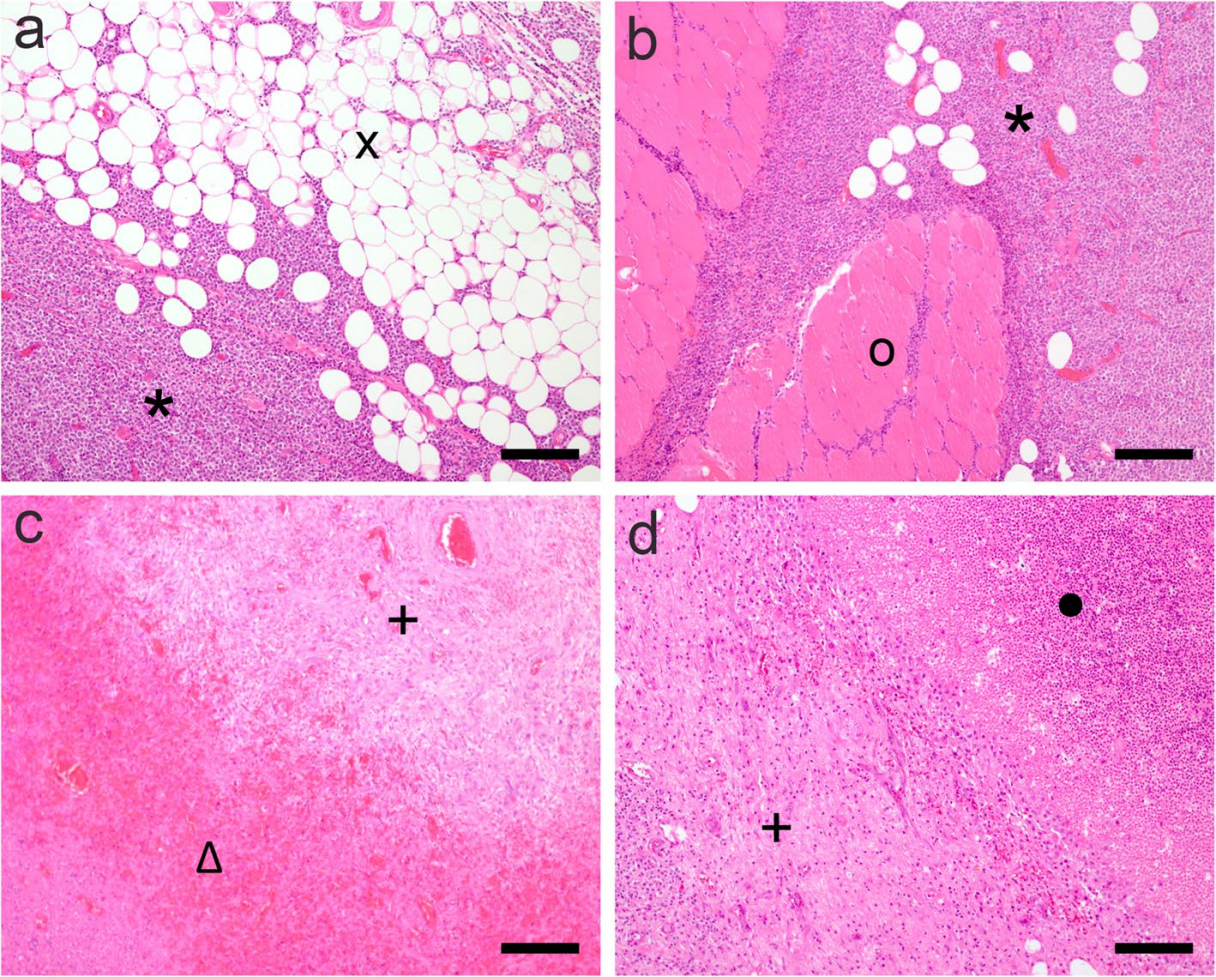
Lymphoma with infiltrative growth into adjacent soft tissue associated with tumor necrosis and hemorrhage. **a** Atypical lymphoid cells (*) severely infiltrate the autochtone adipose tissue (x) (**b**) as well as the skeletal muscle tissue (o) in the mid cervical region. **c** The tumor mass reveals scattered necrotic (●) and (**d**) hemorrhagic (∆) foci demarcated by cell rich, juvenile granulation tissue ( +). Hematoxylin and eosin (HE), bar represents 160 µm

**Fig. 5:**
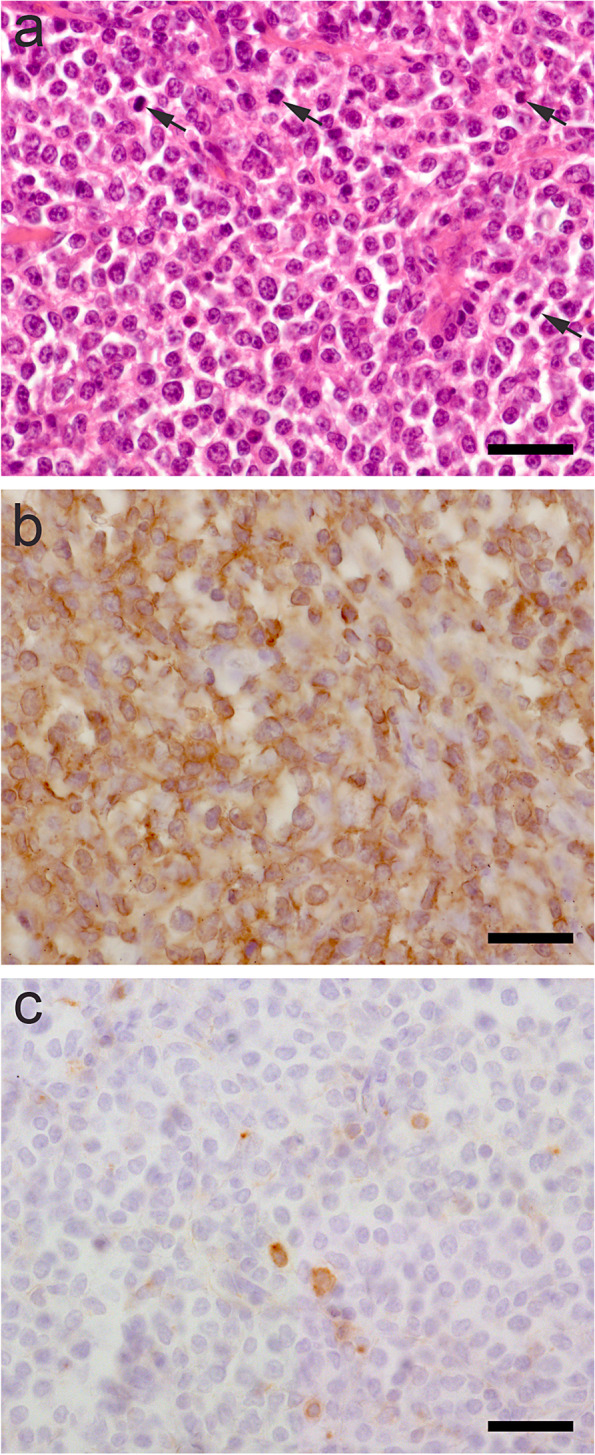
Characterization of tumor tissue by immunohistochemistry performed on paraffin sections. **a** Diffuse proliferation of mildly pleomorphic middle sized neoplastic lymphocytes. The predominantly round shaped, occasionally indented, eccentrically located nuclei harbor granular chromatin with no evident nucleoli and exhibit a mild mitotic activity of 42 mitoses per 10 HPF (HE, representative mitotic figures indicated by arrows); (**b**) The vast majority of neoplastic cells show highly specific cytoplasmic staining for T-cell antigens CD3 (brown). **c** Only single interspersed cells show a specific signal for the B-cell lineage detected by antigen CD79a (brown). Bar represents 30 µm

Histopathological examination of the surgically removed mass was performed. The size of the fixed tumor specimen excised from the middle of the ventral, cervical region was 7 x *6 x *2.5 cm and of firm and elastic consistency. The inhomogeneous cut surface consisted of a beige to yellowish soft tissue with oligofocal, clearly demarcated, irregular formed areas of whitish colour. After fixation for 24 h in 4% neutral buffered formaldehyde, the sample was dehydrated in rising concentrations of ethanol and embedded in paraffin wax. Sections of 4 µm thickness were stained with hematoxylin and eosin (HE) (Figs. 4 and 5a) or conducted to immunohistochemical investigations. For identification of T-cell lineage (CD3) and B-cell lineage (CD79a) in the LabVision-Autostainer (Thermo Fisher Scientific, Fremont, California, USA) antibodies were used against CD79a (M7051 at 1:100 dilution; Dako; Glostrup; Denmark) and CD3 (A452 at 1:100 dilution; Dako; Glostrup; Denmark) respectively. The biotin–streptavidin–peroxidase method was used applied for visualization.

Histopathological investigations confirmed a highly cellular, nodular tumor mass consisting of lymphoid cells accompanied by sparse amounts of fibrovascular stroma (Fig. 4), thoroughly infiltrating adjacent fatty (Fig. 4) and skeletal tissue (Fig. 4). The proliferating cells (Fig. 5a) show mild pleomorphic morphology and exhibit a cell diameter equivalent to the one of 1 ½—2 RBC´s. The tumor cells harbor moderate amounts of eosinophilic cytoplasm surrounding predominantly round shaped, occasionally indented, eccentrically located nuclei. The chromatin structure is granulated, sometimes marginalized, nucleoli are not evident. Mitotic activity was evaluated on HE stained sections by manual counting of mitosis in 10 HPF (high power field). Tumor cells exhibit mild mitotic activity of 42 mitoses per 10 HPF Tingible body macrophages are sparsely distributed throughout the tumor tissue as well as single cells to small cell groups of regular lymphocytes and plasma cells. Moreover, disseminated areas of haemorrhages (Fig. 4c) and necrosis (Fig. 4d) are present throughout the proliferating mass, which are occasionally demarcated by cell-rich juvenile granulation tissue (Fig. 4c).

Immunohistochemical analysis displayed that the neoplasia was composed of diffuse, numerous CD3 immunolabelled cells (Fig. 5b) and interspersed rare, single CD79α-positive cells (Fig. 5c). As a positive and negate control for immunohistochemistry tissue sections from a canine lymph node were analysed in parallel.

## Discussion and Conclusions

Neoplasia are commonly diagnosed in SAC. As their popularity is still ongoing and mostly kept as pets in many countries, they often reach a higher age than other ruminants [12, 13]. This longer life span as well as their rising keeping as pets are probably the reasons for the increased literature reporting on tumors in llamas and alpacas including adenocarcinoma, lipoma, lymphoma, fibroma, fibropapilloma, carcinoma, melanocytoma, leiomyosarcoma and other neoplasia with lymphoma being the most common one [2, 3]. Whereas, lymphoma is the most common malignant neoplasm affecting SAC [1–10]. A study from 2007 indicates that the prevalence of neoplasia is higher in llamas than alpacas. The mean age of llamas with neoplasia (12.53 +—3.2 years) is much higher than in alpacas (5.48 +—3.7 years), whereas SAC with lymphoma had a mean age of a (4.24 +—6.2 years [3, 5].

This is consistent with the age of the patient described, who was 5 years and 9 months old at the time of diagnosis.

Considering age, malignant round cell neoplasms can be differentiated by their immunohistological profiles and locations. T cell lymphoma in SAC have been reported albeit less frequently than B cell lymphomas [8]. Lymphomas in llamas presented as either adult multicentric lymphoma of B-cell origin in animals younger than 7 years of age or T-cell lymphoma and non–B-cell, non–T-cell lymphoma in animals 7 years of age or older [14]. According to literature, several organs can be involved in SAC with malignant round cell neoplasia as a primary lesion or metastasis such as diaphragm, of cardiac and pulmonary structures, uterus and spleen, but liver (65%-80%) and kidney (35%-60%) are the most commonly involved organs [1, 8]. According to Aboellail [14], in contrast to alpacas, the thorax was commonly involved in llamas, with infiltration of neoplastic cells into hilar and mediastinal lymph nodes. T cell lymphomas can also form multinodular periaortic and paravertebral tumor masses [8]. In the case presented here an involvement other organs could not be diagnosed based on clinical and sonographic examination.

In a study investigated 110 camelid neoplasm and diagnosed lymphoma and/or leukemia in 20 alpacas and 6 llamas [14]. In the above mentioned study was reported that some tumors presented a diagnostic challenge because they could not be distinguished based on gross or microscopic morphology. Immunohistochemistry (T-cell marker (cluster of differentiation [CD] 3), a B-cell marker (paired box protein [PAX]-5), a leukocyte integrin beta-2 marker (CD18), and a neuroendocrine marker (synaptophysin) was necessary to differentiate between lymphoma and other malignant round cell tumors (14). Because of the heterogeneous population of malignant round cell tumors it is necessary to distinguish them via immunohistochemistry, as post mortem findings, routine light microscopy and signalment cannot [8, 10]. In our study we used CD3 to detect T-cell specific origin of cells and CD79α antibody was used as a B-cell marker in line with Martin et al. 2009 (8) as well as with Sartin et al. 2004 (10). Martin et al. 2009 mentioned that immunohistochemical studies indicated that CD3 and CD79α appropriately labeled T and B-lymphocytes, respectively, in normal camelid lymph node. Since the tumor in our case revealed highly specific T-cell-positivity in the vast majority of cells, there was from our point of view no further indication to perform CD18 or synaptophysin specific immunohistochemical analysis. In the study of Aboellail et al. 2013, the leucocyte marker CD18 was used to analyze round cell tumors, which lack a relevant B- and T-cell expression of the proliferating cells. In case of high expression of CD18 with concurrent absence of B- and T-cell specific staining the final diagnosis of myeloid leukemia was made in corresponding publication. In the paper of Martin et al. 2009, synaptophysin was used to appropriately label pancreatic islet cells.

Common clinical signs are often nonspecific and highly variable like tissue enlargement in different palpable areas, sizes or shapes, anorexia, weight loss, poor growth, weakness or recumbency [1, 8]. In this case report, the owners called the veterinarian for the dyspnoea and abnormal respiratory sounds. A thorough clinical examination revealed the mass in the cervical area. Blood chemistry profiles displayed similar findings compared to previous studies: severely increased WBC and moderatly increased segmented neutrophils [1], as well as a lymphopenia. However normal lymphocyte counts have been reported as well.

Diagnostic workup associated with neoplasms has been widely described in case reports or case series [1, 8]. During clinical examination, dyspnoea, abdominally reinforced breathing and bilateral dilated nostrils were observed. Ultrasonography of the lung showed retracted lung surfaces and comet-tail-artefacts, whereas focal lesions in the caudal lung lobe. CT scan showed multiple focal bronchocentric speculate nodules. The animal was euthanized due to poor prognosis. Histopathological examination revealed bronchoalveolar carcinoma. Marchionatti et al. [11] present an adult llama, with the history of respiratory problems. The animal showed mild dyspnoea and inspiratory stridor. Ultrasonographic and radiographic examination detected a soft tissue mass in the tracheal wall, which markedly decreased the air-filled tracheal lumen. For a more detailed evaluation of the mass and connection between neighbouring tissues a fine needle aspiration and following cytological examination, as well as a CT scan was carried out. It revealed a solitary tracheal B-cell lymphoma localised 30 cm caudal to the larynx and attached to the tracheal serosa. Due to poor prognosis, it was euthanized. In a series of 12 cases of malignant round cell tumors ultrasonography of 6 animals detected evidence of neoplasia in 4 cases and biopsy of enlarged lymph nodes or liver resulted in 5 out of 5 diagnoses of neoplasia [5]. The latter cases, as well as this case report point out, how important diagnostic imaging and fine needle aspiration or biopsy and following cytological examination, additionally to the clinical examination and laboratory tests, are for a detailed evaluation and to distinguish between neoplasia and other differentials (e.g. tuberculosis, fungal granulomas). Unfortunately, there are only a few described examinations techniques for different diagnostic imaging in SAC [15–17]. Cytology of the mass (i.e. FNA) was not performed in this case before the surgery. FNA would have given definitely a quite accurate preliminary diagnosis helping with the surgical planning and approach to the mass and should be done in following cases. Additionally, there is a lack of descriptions of physiological findings or pathological findings based on diagnostic imaging in SAC [15–20].

During clinical examination and diagnostic imaging no metastasis or lymphadenomegaly could be found, although lymph node enlargement is a common symptom [1, 10], more often found during necropsy than physical examination [5]. Whereas Cebra et al. [18] reported, that clinical courses in SAC are often short probably because of the advanced stage prior to diagnosis. A routinely performed thorough evaluation of peripheral lymph nodes during clinical examinations may lead to an earlier detection of malignant round cell tumors [5].

This study and previous studies indicate that camelids with MRCT can present with a variety of clinical signs and laboratory findings, and that the clinical course is variable. This case report points out, that an early detection followed by surgical procedures can expand the life span of the affected animal.

## Data Availability

All data of the case are available by the corresponding author.

## Abbreviations

CT: Computer tomography
e.g.: For example
CD: Cluster of differentiation
PAX: Paired box protein
BCS: Body condition score
SAC: South American Camelids
